# Atopic dermatitis in Tunisian schoolchildren

**DOI:** 10.4314/pamj.v9i1.71210

**Published:** 2011-07-28

**Authors:** Meriem Amouri, Abderahmen Masmoudi, Nozha Borgi, Ahmed Rebai, Hamida Turki

**Affiliations:** 1 Department of Dermatology, Hedi Chaker Hospital, Sfax, Tunisia; 2 National office of Family and Population, Sfax, Tunisia; 3 Center of biotechnology, Sfax, Tunisia

**Keywords:** Atopic dermatitis, atopy, children, epidemiology, prevalence, Tunisia

## Abstract

**Introduction:**

The prevalence of atopic dermatitis (AD) is low in North Africa. We describe the epidemiology of this atopic condition among school children in Tunisia.

**Methods:**

We conducted a Cross-sectional survey study of 5 to 6-year-old schoolchildren from 21 primary schools of Sfax. The diagnosis of AD was based on the U.K. Working Party diagnostic criteria. A questionnaire including these criteria and some risk factors of AD was issued to the children. All children were examined by one dermatologist.

**Results:**

Among the 1617 examined children, ten had AD giving a one-year prevalence of 0.65%. The overall sex ratio was 2.33. The disease occurred before the age of 2 years in 3 children. Pure AD without concomitant respiratory allergies was noted in 3 cases. One first-degree family member with atopy was at least noted in seven children. The strongest associated factor was the presence of AD in at least one parent and maternal age at the time of the child birth. Nor breast-feeding neither environmental characteristics of the house did correlate with AD.

**Conclusion:**

The prevalence of AD in Tunisian schoolchildren is low but comparable to those of other developing countries. Family history of atopy and maternal age at the birth time was the most important associated factors.

## Introduction

Atopic dermatitis (AD) is a common inflammatory skin disease among children. Most of the studies on the epidemiology of AD are based on the Western population, mainly in UK and Northern Europe [1]. The prevalence of AD appears to have increased over the past three decades in Western countries, affecting 2-3% of children before 1960 to 9-12% in those born after 1970 [2]. However, only few studies have addressed this issue in countries with a temperate climate, including those of the Mediterranean area [3]. The International Study of Asthma and Allergies in Childhood (ISAAC) study, based on self-report questionnaires, is the most important of them [4].

Tunisia is a North African country with almost 10 million of population. The aim of this study was to estimate the prevalence of AD in Tunisian schoolchildren from the region of Sfax. It is an industrial city and the 2nd most crowded city of Tunisia. It accounts 931 000 habitants in year 2010. The insolation is relatively long and minimal relative humidity is below 30%. The sirocco, which blows mainly in summer, increases photochemical pollution, hinders the dispersion of pollutants and directs them towards the most crowded areas of the city.

The schoolchildren were examined by a single dermatologist. We used a standardized questionnaire to elicit information on associated allergic diseases, characteristics of the family and other environmental factors. The diagnosis of AD was based on the Specificity on the UK Working Party criteria.

## Methods

### Sample design and recruitment

The population study consisted of schoolchildren attending the first grade elementary school (aged between 6 and 7 years old). It was carried out between the 14^th^ of February and 7^th^ of March 2007 in Sfax city. After ethics committee and school authorities permission, one of us (A.A.M.) visited all schools in the city. The children were first examined then given a one-page questionnaire to be completed by the parents. The same person ensured that the questionnaires were duly completed and collected. Children who were absent on the day of examination, twins and those aged less than 6 years old or older than 7 years were excluded from the statistics. The aim of examination was to look for flexural eczema (minor criteria).

### Diagnostic criteria and questionnaire

AD was defined using the U.K. Working Party diagnostic criteria for AD [1]. Asthma and allergic rhino-conjunctivitis (A.R.C) were defined, according to those criteria, respectively as “bouts of wheezing or coughing” and “bouts of sneezing with runny nose or itchy eyes in summer”. The French version of these criteria [5] was translated into Arabic then back translated in to French (second most spoken language in Tunisia). It was designed to elicit demographic (age of the child and the parents, sex, place of birth), socioeconomic (as reflected by the occupation of the parents), personal and family history of atopic disorders. Perinatal variables (duration of breast-feeding and age of introduction of solid food) were recorded. Environmental factors such as smoking habits of the parents, type of housing (apartment, villa or patio open house), domestic animals, number of siblings, number of persons per room and carpets at home were asked about.

### Statistical analysis

All data were analysed using the Statistical Package for the Social Sciences version 11.0 for Windows. The analysis included all the variables listed previously. Continuous variables were analysed by t-student test and comparative variables were analysed with the chi-square test. A p value less than 0.05 was considered statistically significant.

## Results

### Prevalence of AD, asthma and RC

We excluded 95 children from the statical analysis: children aged beyond seven or less than six years old (27 cases), twins (48 cases) and who were not examined (20 children). One thousand three hundred and forty nine children out of 1522 gave back the questionnaire. The overall response rate was 88.6%. AD was diagnosed in ten of the 1522 examined children, with a one year-prevalence of 0.65%. The term "one-year prevalence" means that we asked about “An itchy skin condition in the last 12 months”.

The reported lifetime prevalence of asthma and ARC in the children with AD were respectively 33.33% and 70% whereas they were 10.13% and 6.87% in the children without AD. The difference was statically significant for ARC (p≤0,001) but not for asthma (p=0,056). Three children had the three allergic diseases together.

### Descriptive epidemiology

Among the 1522 examined children, there was 793 boys and 729 girls (sex ratio 1.08). Among the AD group, there were more boys than girls (sex-ratio 2.33). The strongest predictor factor for AD was the presence of a history of flexural dermatitis on skin creases, followed by the history of dry skin in the last year or of RCA. Onsets under the age of 2 years and the history of asthma were less strongly associated with AD. Visible flexural dermatitis was seen in 5 children with AD (Table 1). The principal site of eczema was the Knees (4 cases) followed by the elbows (2 cases). The ankles and around the eyes were affected each in one case. No localisation around the neck was found.

**Table 1:** Number of children with individual features used in the UK diagnostic criteria for atopic dermatitis

UK Working Party's criteria	Number of children
An itchy skin condition in the last 12 months	10
Onset below age 2	3
History of flexural involvement or skin creases	10
History of a generally dry skin	7
	
**Personal history of other atopic disease**	
Asthma	3
Asymptomatic rhinoconjunctivitis allergica (RCA)	7
Visible flexural dermatitis	5

For children with other atopic diseases, Asthma started at the same time, before or after AD each in one case. However RC stared at the same time in two cases, before in three cases or after AD in one case.

### Analytic epidemiology

#### Familiar Atopic diseases

Six children (60%) had at least one first-degree family member with atopy among children with AD. Respectively, four (40%), three (30%) and four (40%) children with AD had at least one relative with AD, asthma and ARC while only 8.57%, 8% and 11.41% of unaffected children had. The difference was statistically significant (respective p value 0.008, 0.033 and 0.014) (Table 2).

**Table 2:** Higher lifetime prevalence of atopic diseases in relatives of atopic dermatitis (AD) index cases than in relatives of unaffected controls

Familial history of atopic diseases	AD	Asthma	ARC
Without AD	111 (8.57%)	105 (8.2%)	146 (11.41%)
AD	4 (40%)	3 (33.33%)	4 (40%)
P value	0.008	0.033	0.014

RCA: asymptomatic rhinoconjunctivitis allergica

#### Potential risk factors

The average paternal age of the children with AD was 44.6 +/-5.35 while it was 41.83 +/-5.38 for the children without AD. The difference was not statistically significant (p=0.13). Maternal age ranged between 35 and 45 years (39.1 +/-2.96 years) in the AD group while it was 35.4 years (+/-5.14). It was significantly associated with AD (p=0.003).

All the children with AD were from Sfax city. The type of housing did not correlate with occurrence of AD. Respective p values were (0.72 and 0.29). Living in a patio open house showed a trend towards an association with AD (p=0.056).

No association was found with the child's range among sibling, number of sibling, housing density and age of introduction of solid food (Table 3). Parental smoking, breast-feeding, presence of domestic animals and carpets at home were not associated with AD (respective p values p=0.5, 0.33, 0.26 and 1).

**Table 3:** No association with familial, environmental or nutritional factors

Risk factors	AD	No AD	P value
Range of the child			
2^nd^ place	7	455	1
3^rd^ place	3	219	1
Number of sibling			
1	6	611	0.759
2	3	384	0.938
3	1	79	0.461
Housing density	1.53	1.39	0.54
Age of introduction of solid food (months)	7.2	8.06	0.38

## Discussion

AD is a chronic, relapsing skin disease characterized by pruritus, a predilection for the flexural areas, and a personal or family history of atopy. The UK working party's criteria consist of reliable discriminators for AD, using the Hanifin and Rajka list of clinical features as the building blocks [1]. The present study is the first using these criteria in Tunisia.

The point prevalence was 0.65% as assessed by the UK working party criteria in 6 to 7-year-old children in 2007. All the other Tunisian studies confirmed this low prevalence of AD [2-4]. A multicentric retrospective study in 7 dermatological departments in Tunisia carried between 1996 and 2000 found an incidence of 0.52 (with variations between 0.01 and 1.02) [2]. This is similar to that reported by another comparative study of the prevalence of AD in Tunisia and France which found a prevalence of 21% in France and of 0.44% in Tunisia. Thus, the risk of having an AD for a French child is 49.3 times that of a Tunisian one [3]. This general low prevalence of AD in Tunisia is in concordance with the others developing countries [4]. However the ISAAC study found a higher prevalence among 13-14 years old schoolchildren with an increase from 8 to 9.4% in 5 years [6]. ISAAC is a questionnaire-based study and the children are not examined. Similar high prevalence of AD was seen in Algeria, Morocco, Oman and Kuwait [7]. The high prevalence of AD among school children in Tunisia is far from that observed in developed countries, such as the U.K., northern Europe, Australia, Japan and Hong Kong. This suggests that environmental factors may be important in determining disease expression. In addition, constitutional, genetic and epigenetic factors may play a role in this particular epidemiology of AD. Thus, they have to be elucidated by intensive researches in Tunisia.

To our mind, the response rate (88.6%) did not influence the real prevalence rate. It has been reported that low response rate over estimates the prevalence, as children with cutaneous complaints might be more likely to participate in the study than children without it [8].

AD started before the age of two in only 3 cases. This late onset is common in Tunisia. kharfi et al. found a medium age onset of 3.2 years old [9]. In Singapore, where the prevalence of AD is high, about half the children at the age of 16 developed symptoms of AD in the first 10 years of life (49.5%).

Our study shows that 6 to 7 years old boys are more affected than girls. While previous studies showed approximately an equal sex ratio for AD or a female preponderance. Mohrenschlager and coll. showed, by measuring the pH and hydratation degree of the stratum corneum, that girls aged between 7 and 9 years old are more likely to develop AD [10]. In fact, the sex ratio depends on the age. AD is more common among boys in the younger age groups and girls during the adolescence [4]. Life style, such as the washing habits or the use of cosmetics, is also incriminated in this difference.

Only three boys had pure AD (30%) while the seven other children (70%) had a mixed type with concomitant respiratory allergies. This can be compared with the study carried out by Diepgen and Fartasch where 54% had pure AD and 46% suffered from a mixed type [11]. The “atopic march” postulates that a child progresses from AD to asthma and ARC as he/she becomes older. Our results do not confirm this notion as asthma started indifferently as to AD and ARC started before AD in 3 cases. It has also been noted previously that the epidemiology of AD seems to be much more aligned to ARC than asthma [2,12].

A higher lifetime prevalence of atopic diseases (AD, asthma and ARC) was found in the relatives of children with current AD than in the relatives of unaffected controls, as reported in many studies focusing on the familial association of atopy. Since information was obtained by history taking, accuracy of recall should remain a point of concern. The real importance of a family history of atopy as a risk factor for the development of AD is well documented in cohort studies. Seven children with AD had at least one family member with atopy. The proportions of AD and ARC were equal in the family members (40%) and more than that for asthma (30%). In the study by Larsen et al., 59% of the subjects had a family history of atopy and the proportion of family members with AD and ARC were equal and twice that of asthma [8].

Although cohort studies are also better suited to ascertain risk factors, our cross-sectional survey was designed to collect information at least on factors that were not modifiable on the basis of the child status, such as prenatal and perinatal variables. None of the variables tested were associated with AD as already suggested in the literature.

The prevalence of asthma and ARC were high, 10.13% and 6.87%, respectively. The rapid urbanization of Sfax city, its westernized life-style, the high standard of living and education may account for this frequency. This trend is in accordance with those in developed countries.

Our study indicates that AD is very rare in Tunisian schoolchildren. Many difficulties arise while directly comparing findings across studies, because the estimates have been shown to be dependent upon the disease definition, methods of assessment, age ranges, year of birth of the children studied or period of time in which the study was conducted and geography [8].

The UK working party criteria are repeatable, simple and applicable to different ages and ethnic groups. They showed very good sensitivity and specificity, with high positive and negative predictive values when compared to clinical diagnosis in many populations and are confirmed to be a practical and reliable epidemiological tool in the investigation of AD in population settings [1]. However, these criteria have not been validated in Tunisia. Thus we have no idea whether they are suitable for Tunisian children or not. In fact, many previous studies showed a low sensitivity of these criteria mainly when AD is rare [13,14].

Chalmers et al. explained the low validity of the U.K. diagnostic criteria by a combination of translational and cultural issues. The authors suggested that the one physical sign of visible flexural eczema may be useful for future international comparative prevalence studies [14].

## Conclusion

In conclusion, our study is the largest and the most comprehensive done in Tunisia to date. The prevalence of AD is low in Tunisia by using the UK working party criteria. Family history of atopy and maternal age at the birth time are statically associated with AD. However, we used non validated criteria in Tunisia which might have affected the real prevalence of AD in our country.
